# Remarks on a Bill for Repealing the Act Relative to Lunatics Now in Force, and Substituting Another in Its Stead

**Published:** 1814-07

**Authors:** 


*For the Medical and Physical Journal.*

### *Remarks on a Bill for repealing the Act relative to Lunatics now in force, and substituting another in its stead.*

I TAKE the liberty of submitting to the public, through your means, a few remarks on a bill which has been lately brought into parliament, by Mr. Rose, for the purpose of repealing the act relative to lunatics at present in force, and substituting another in its stead.

The licensing and inspection of private lunatic hospitals in London and its environs, and in Middlesex, attach to five commissioners, annually chosen from the College of Physicians. In other parts of England, the licenses are granted by the quarter sessions of the peace, and the inspection takes place by a board, annually appointed by such quarter sessions, consisting of two magistrates and one physician. The general provisions are very much the same in both acts, and and are enforced by appropriate penalties. But there are a few alterations in the present bill, one of which is of particular importance, viz. that persons having one lunatic only confined in a house, must give in to the commissioners the name of such individual, who becomes, after a certain period, liable to inspection, from which he is at present free. The other alterations are upon the whole improvements, though perhaps not to the practical extent which may at first view be imagined.

But there are some points in this bill which give it a much more leading and prominent characteristic than any which concern the patient. By the act at present in force, houses which do not contain more than one, and not exceeding ten persons, pay 10*l.* per annum for their license; and all which contain above ten, pay 15*l.* per annum, with a fee, in both cases, of 6*s.* 8*d.* to the secretary. This fund is placed under the charge of the treasurer of the College of Physicians, who is out of it to pay one guinea for each visitation, to each of the commissioners who may attend it, three of them being a quorum; and who is also to defray the other expences of such visitation, and to pay to the secretary such an allowance for his trouble as may be determined by the commissioners. The same general plan is pursued in the country, the clerk of the peace being secretary and treasurer.

Now I beg you to remark the alterations proposed in the present bill. Instead of paying a sum, which never exceeds 15*l.* per annum, for their license, the keepers of lunatic houses must pay 10*l.* per annum for every number above 1, and below 10, and 5*l.* for every additional 10 up to 100, and then 5*l.* for every additional 20; parish poor being chargeable with one-half of the sum here stated. Hence, if it is supposed that the lunatic houses in London and its neighbourhood average 60 patients each, which I presume is not an immoderate average, then the keeper must pay 40*l.* for his license instead of 15*l.* But this is not all: every keeper of a lunatic house, under the penalty of 100*l.* and the forfeiture of his license, is required to transmit, with the other notices, to the Secretary of the Commissioners, three days after a patient's admission, an account of "the weekly, or other stipend or payment" to be made to him for the maintenance of every such patient; in order that he may pay, to the College of Physicians, "such sum of money as the annual or other pay or stipend agreed to be paid to such keeper or keepers for the care and maintenance of every patient to be received therein, shall amount unto for two weeks." By this means, every lunatic house keeper, besides paying for his licence almost three times the annual sum which he has heretofore done, must actually be charged to the College, for the use of that body, and the payment of the expences of the commission, the 1-26th part of the gross sum received by him in the course of his business, without any reference to his profit. This supposes that a patient may be in a lunatic house a whole year; but, as the clause is worded, it will even require a fortnight's pay, or stipend, for however short a period he may be so confined. At the probable amount of this tax it is very difficult to guess; but if you take the number 60 as an average, and suppose that 40 of those are patients who pay 10*s.* 6*d.* per week, and the remainder, patients who average a payment of 1½ guinea per week, (which I take it is a very low average, since there are many who pay from that sum up to 10 guineas,) it will follow, that 105*l.* must be annually paid for 60 patients; which, when added to the sum charged for a license, produces the enormous increase of 120*l.* instead of 15*l.* per annum, the whole present expences. But this sum, great as it is, does not take into account the fortnight's expence of all such as are for a short period only confined in a lunatic house, and of such as are in separate houses, and who must therefore pay, as is above stated, not only separate license fees of 10*l.* but a fortnight's amount of whatever their pay or other stipend may be.

It does not appear that any case has been made out, or even attempted to be made out, for such a serious tax upon lunatics; for it is clear that it is by lunatics or their friends, and not by the keepers of houses, that this tax must eventually be paid. The preamble to the bill states, that the existing act has been found insufficient for the purposes intended by it, without in any way pointing out its insufficiency, or offering against it any ground of complaint. And yet it is difficult to conceive what practical purpose the present bill, if made into a law, will more effectually answer than the former one, except the augmenting, in a most extraordinary degree, the expences attending it, for the benefit of a private corporation. The business of the existing act has been done, for 40 years, at its present rate, and a moderate augmentation to the fees of the commissioners, and to the license money which is to defray them, is very proper; for the labourer is worthy of his hire; and there is no class of men more distinguished for liberality in the practice of their profession, than are physicians. But Mr. Rose's bill has an evident tendency to throw a slur upon that part of the body of physicians (a small one only) which is to reap the pecuniary advantages of the enactments provided by it: for it conveys the degrading idea, that, in the performance of a public duty entrusted to them by the legislature, those gentlemen, contrary to what I am sure is the fact, are principally actuated by motives of pecuniary advantage.

I have looked in vain into the new bill for any check upon the proceedings of the commissioners. The bill provides, that the magistrate and physician who are to act as the inspecting committee in the country, are to take minutes of their proceedings, which shall be entered in a register, and that such register shall be open to inspection, at all seasonable hours, at the office of the Clerk of the Peace; but from any such inspection the records of the London Commissioners are to be free: and the very small portion of publicity which the existing act gives to any matter of reprehension, by requiring that the minute relative to it should be hung up in the Censor's room of the College, is not provided for at all in the present bill. But there is one other point to which I must beg to draw your attention in the present bill; it is this, that the framer of it, by a side wind, attempts to bring every country physician within the pale and jurisdiction of the College of Physicians of London, and thus to increase the powers and emoluments of that body: for he makes it necessary, that in order to be an inspecting physician, and even to be able to sign a certificate of a patient's insanity, a circumstance of continual occurrence, a physician must have been previously examined and approved by the College of Physicians, unless he should have been a graduate of Oxford and Cambridge. Now it must be admitted, that, though Oxford and Cambridge deservedly possess high reputation as seminaries of general literature and science, they have no pretensions whatever, as medical schools; and hence it is, that 19 in 20 of the country practitioners, and most of those in London, have obtained their education in other universities, and particularly in that of Edinburgh. Many of our provincial physicians are, and have been, among the most eminent practitioners of this country, without being connected with the College; and considering how much better education, both general and professional, is at present than formerly, considering likewise that this law, though it has existed for nearly 300 years, has never been enforced, if indeed it is now any thing else than a dead letter, it seems to be very singular that an attempt should at this time be made, to give such a law action, by indirect means.

If it is thought proper to make country physicians amenable to the College of Physicians of London, instead of being satisfied with the discipline of the universities where they may have taken their degrees, why should this not be proposed to the legislature in the form of a statute, instead of being foisted into a bill for a very different purpose?

I trust

I trust that these remarks will receive attention from the professional public, particularly country physicians. Such a bill as is the proposed one, is hardly likely to pass; but if it succeed in advancing to another stage in the House, those gentlemen would do well to petition against it, from different districts. X. Y. Z.

